# The Roles of Picornavirus Untranslated Regions in Infection and Innate Immunity

**DOI:** 10.3389/fmicb.2018.00485

**Published:** 2018-03-20

**Authors:** Anna Kloc, Devendra K. Rai, Elizabeth Rieder

**Affiliations:** Foreign Animal Disease Research Unit, Plum Island Animal Disease Center, Agricultural Research Service, United States Department of Agriculture, Greenport, NY, United States

**Keywords:** picornaviruses, 5′- and 3′-UTRs, RNA functional elements, foot-and-mouth disease virus (FMDV), modulation of innate immunity, RNA viruses

## Abstract

Viral genomes have evolved to maximize their potential of overcoming host defense mechanisms and to induce a variety of disease syndromes. Structurally, a genome of a virus consists of coding and noncoding regions, and both have been shown to contribute to initiation and progression of disease. Accumulated work in picornaviruses has stressed out the importance of the noncoding RNAs, or untranslated 5′- and 3′-regions (UTRs), in both replication and translation of viral genomes. Unsurprisingly, defects in these processes have been reported to cause viral attenuation and affect viral pathogenicity. However, substantial evidence suggests that these untranslated RNAs may influence the outcome of the host innate immune response. This review discusses the involvement of 5′- and 3′-terminus UTRs in induction and regulation of host immunity and its consequences for viral life cycle and virulence.

## Introduction

Viral–host interactions dictate the progression of disease. To successfully infect host cells, picornaviruses evolved an extensive repertoire of methods to enter a host cell, replicate their genome, and disarm the host defense mechanisms. The latest include: (i) shutting down host protein synthesis, (ii) interfering with the recognition of pathogen recognition receptors (PRRs), and (iii) disrupting the host innate immune system signaling cascades. Many of these tasks are accomplished by viral proteins, which–through interactions with both viral and host proteins – facilitate infection. A telling example are picornavirus proteinases, such as 2A^pro^, L^pro^, and 3C^pro^ proteins, which are important for processing of the viral polyproteins. In addition, 2A^pro^ has been shown to antagonize the host immune response by cleaving melanoma differentiation-associated 5 (MDA5), mitochondrial antiviral signaling (MAVS), and TIR domain-containing adapter-inducting interferon-β (TRIF), which inhibits IFN-β and type III interferon responses (Wang et al., 2013; Feng et al., 2014; Lind et al., 2016; Lei and Hilgenfeld, 2017), and degrades poly(A)-binding protein (PABP) and eukaryotic initiation factor 4G (eIF4G) to shutoff host translation machinery (Kerekatte et al., 1999; Glaser and Skern, 2000). On the other hand, 3C^pro^ cleaves interferon regulatory factors (IRFs) (Lei et al., 2013), TRAF family member-associated NF-κB activator (TANK) (Wang et al., 2015; Huang et al., 2017), inhibitor of kappa B kinase γ (IKKγ) (Wang et al., 2012, 2014), and inhibits the innate immune system cascades by modulating retinoic acid-inducible gene I (RIG-I), MDA5, and MAVS (Papon et al., 2009; Qian et al., 2017; Rui et al., 2017). The foot-and-mouth disease virus (FMDV) L^pro^ degrades the p65/RelA subunit of NF-κB (de Los Santos et al., 2007, 2009) and binds ADNP (host transcription factor), which can interfere with the expression of IFNs and interferon stimulated genes (ISGs) (Medina et al., 2017).

Research and data obtained from analyses of clinical and field samples provide ample evidence that the noncoding regions of the picornavirus genome can contribute to viral pathogenicity. Their 5′- and 3′-UTRs fold into many secondary and tertiary structures due to base pairing, giving rise to stem–loops (SLs), clover-like structures, or pseudoknots (PKs), as well as internal ribosome entry site (IRES) elements (Martinez-Salas et al., 2018). Point mutations, deletions, or insertions in these regions have been shown to be associated with attenuated phenotypes *in vivo*, and lower viral replication and translation rates. While some of these structures are already known to interact with host proteins, additional motifs and/or specific sequences present within these noncoding elements may influence both recognition of viral elements by host PRRs and activation of the host innate immune response system. Taken together, the above examples illustrate a remarkable structural/functional plasticity of picornaviruses and the ability of these viruses to counteract the host antiviral strategies.

## Picornaviridae Genome Composition

**FIGURE 1:**
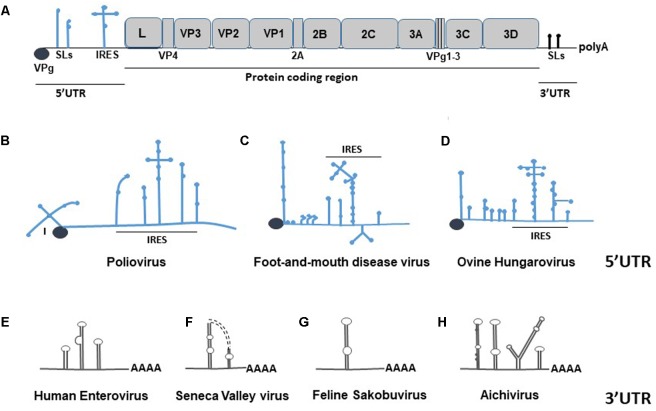
Schematic representation of a picornavirus genome. **(A)** Depiction of FMDV genome, where the RNA elements of the 5′- and 3′-UTRs are drawn in blue and black, respectively. The protein coding region of the genome is depicted as gray boxes, which correspond to individual genes. **(B–D)** Examples of secondary structural variation within the 5′-UTR region of different picornaviruses. The types/numbers of loops and the IRES are drawn in blue. **(E–H)** Examples of picornavirus 3′-UTR secondary structure diversity, shown in black.

Picornaviruses comprise a large group of viruses that cause a variety of human and animal diseases, including respiratory infections, paralysis, hepatitis, and meningitis (Atmar et al., 2012; Tapparel et al., 2013). Currently, the International Committee on Taxonomy of Viruses (ICTV) identifies 35 genera of picornaviruses, with 24 genera consisting of a single viral species (**Table 1**). Picornaviruses are strictly cytoplasmic viruses, meaning that all processes following the viral entry (viral translation, replication, and assembly of viral RNA/proteins) occur in the host’s cytoplasm. Picornavirus genome (ssRNA+) ranges from 6.7 (Aquamavirus) to 9.9 kB (Sicinivirus) and it functions as an mRNA. For example, the FMDV genome gets translated from the second of two in-frame AUG codons, resulting in a single polyprotein that is processed by viral encoded proteinases leader (L^pro^), 3C^pro^, and the peptide bond skipping 2A into the mature structural and nonstructural (NS) proteins. FMDV codes for 4 structural proteins and 10 NS proteins. Viral RNA replication in infected cells is a two-step process carried out primarily by the viral RNA-dependent RNA polymerase (3D^pol^), in conjunction with other viral or cellular proteins. The RNA is transcribed into complementary minus strands, which are then used as templates for the synthesis of the progeny plus strands via a multi-stranded replicative intermediate (RI) complex. The negative-sense RNA serves as a template for the synthesis of multiple copies of genomic RNA, some of which are translated and others that become packaged into virus particles. Due to the lack of proofreading activity of the polymerase, errors are frequently generated during replication and every new genome contains approximately 10^-4^ substitutions per nucleotide (nt) (Escarmis et al., 2008). Therefore, the virus population consists of quasispecies; a collection of genetically diverse members that can rapidly adapt to new environments by selection. The single open-reading frame (ORF) of a picornavirus genome is divided into three regions: P1, which encodes structural proteins, and P2 and P3, which encode viral NS proteins (**Figure 1A**). The ORF is flanked by 5′- and 3′-UTRs. The genomic RNA of picornaviruses is linked to a viral protein genome-linked (VPg) at its 5′-terminus, which acts as a primer during viral RNA synthesis.

**Table 1:** Known untranslated structural features within the *Picornaviridae*.

Picornavirus genus	Representative virus examples	Specific 5′-UTR features	Specific 3′-UTR features
Ampivirus	Ampivirus A	IRES	N/A
Aphthovirus	Bovine rhinitis A	Hairpin SL secondary fragment, poly(C) track, series of pseudoknots (PKs) (except in bovine rhinitis viruses), IRES, *cre* in FMDV, PPT in FMDV	Two SLs (SL1 and SL2) structures in FMDV
	Bovine rhinitis B		
	Equine rhinitis A		
	Foot-and-mouth disease (FMDV)		
Aquamavirus	Seal picornavirus	Hairpin SL, PPT, IRES	N/A
Avihepatovirus	Duck hepatitis A virus	IRES, PPT	Less than four hairpin loops predicted
Avisivirus	Avisivirus A, avisivirus B and C (chicken picornavirus 2&3)	IRES, PPT	N/A
Cardiovirus	Encephalomyocarditis virus (EMCV)	Poly(C) track in EMCV, PKs in EMCV, IRES, PPT	Three SLs in mengovirus


	Theiler’s murine encephalomyelitis virus (TMCV)		
Cosavirus	Cosavirus A1	IRES	N/A
Dicipivirus	Canine picodicistrovirus	IRES, PPT	N/A
Enterovirus	Coxsackievirus (CVB)	Cloverleaf PV, a second “cloverleaf-like structure” in enterovirus E and F, IRES, PPT	Variable; two hairpin SLs in PV, three SLs in CVB B4, on SL in human rhinovirus 14
	Poliovirus (PV)		
	Bovine enterovirus 1		


	Rhinovirus		
Erbovirus	Equine rhinitis B virus	IRES	N/A
Gallivirus	Gallivirus A	IRES, PPT	Multiple SLs predicted, 48 nt “barbell-like structure”


Harkavirus	Falcovirus A1	Cloverleaf, PPT, IRES	SL1 and SL2 predicted
Hepatovirus	Hepatitis A	5′-SL, IRES, PKs, PPT	N/A
	Hepatovirus C		
	Hepatovirus D		
Hunnivirus	Bovine hungarovirus	SLs, IRES, PPT	Two SLs predicted
	Ovine hungarovirus		
Kobuvirus	Aichivirus A	IRES, PPT	Multiple SLs predicted, Aichivirus A is predicted to have a long barbell-like structure


	Kagovirus 1		


	Bovine kobuvirus		
Kunsagivirus	Kunsagivirus A	IRES	Single SL predicted
Limnipivirus	Bluegill picornavirus	5′-Terminus SL in bluegill picornavirus, IRES, PPT	Poly(C) track in bluegill picornaviruses


	Carp picornavirus		
Megrivirus	Turkey hepatitis	IRES	N/A
	Duck megrivirus		
Mischivirus	Mischivirus A, B1, C1	IRES	Multiple SLs predicted
Mosavirus	Mosavirus A1	5′-SL predicted, IRES, PPT	N/A
Oscivirus	Oscivirus A	IRES	N/A
Parechovirus	Human parechovirus	IRES	Single SL in human parechovirus, two SLs in Ljungan virus


	Ljunganvirus1		


	Sebokele virus		
Pasivirus	Pasivirus A	IRES	Single SL swine pasivirus predicted
Passerivirus	Passerivirus A	IRES	N/A
Potamipivirus	Eel picornavirus	IRES	N/A
Rabovirus	Rabovirus A	5′-SL, IRES, predicted PKs, PPT	N/A
Rosavirus	Rosavirus A1	Predicted cloverleaf structure, IRES, PPT	Predicted to form multiple SL structures


Sakobuvirus	Feline sakobuvirus	IRES, predicted PKs, PPT	SL with a “barbell-like” structure
Salivirus	Salivirus A	Predicted SLs, IRES, PPT	N/A
Sapelovirus	Avian sapelovirus	Predicted SLs, IRES	Three SLs predicted in porcine sapelovirus


	Porcine sapelovirus		


Senecavirus	Seneca Valley virus	IRES, two additional SLs predicted	Two SLs predicted to form a “kissing-loop” structure
Sicinivirus	Sicinivirus A	Predicted SLs at 5′-UTR, IRES, PPT	Two SLs, “barbell-like” structure
Teschovirus	Teschovirus A	IRES	N/A
Torchivirus	Tortoise picornavirus	IRES	N/A
Tremovirus	Avian encephalomyelitis virus	5′-SL, two PKs, PPT, IRES	Three SLs predicted

*The currently known 5′ and 3′ UTR features are listed along with the genus and specific strain they are described in. SL, stem loop; PPT, polypyrimidine track.*

The 5′-UTRs of picornaviruses range between 415 nts (Passerivirus) (Woo et al., 2010) and 1,451 nts (Cardiovirus) (Duke et al., 1992), which, depending on a viral species, can comprise up to 17% of the genome (**Table 1**). The 5′-UTR has a higher G+C content compared to the rest of picornavirus genome, which is important for stability of the secondary structures and adaptation to environment (Tapparel et al., 2007). Besides, the 5′-UTR region is characterized by a high degree of sequence homology among picornavirus species. For example, related rhinoviruses have over 60% of homology within the first 600 nts of the 5′-UTR (Stanway, 1990), whereas FMDV isolates share 80–85% nucleotide identify for the S and L fragments of the 5′-UTR (Carrillo et al., 2005). Structures present in the 5- UTR of picornaviruses are important for many events of the viral life cycle. For example, accumulated evidence revealed the importance of the poliovirus (PV) 5′-UTR for viral replication, translation, viral–host protein interactions, and virulence (Andino et al., 1990; Rohll et al., 1994; Rieder et al., 2000; Barton et al., 2001; Lyons et al., 2001; Vogt and Andino, 2010). All *Picornaviridae* have an IRES in their 5′-UTRs, which is important for cap-independent recruitment of the host translation machinery (reviewed in Martinez-Salas et al., 2015). Other structures commonly found in the 5′-UTRs of *Picornaviridae* include SLs, a cloverleaf structure, and PKs (**Figures 1B–D**). The cloverleaf, first described in PV, is a *cis*-acting RNA replication element required for initiation of negative- and positive-strand synthesis (Andino et al., 1990; Rohll et al., 1994; Rieder et al., 2000; Barton et al., 2001; Lyons et al., 2001; Vogt and Andino, 2010). Deleting its fourth nucleotide destabilizes viral RNA and causes a pronounced decrease in the synthesis of the negative strand (Barton et al., 2001). Some members of *Picornaviridae*, such as mengovirus or FMDV contain a poly(C) track near the 5′-terminus, which has been shown to be important for virulence (Duke et al., 1990) and viral growth (Rieder et al., 1993), respectively. The 5′-end of many genera of picornaviruses folds into SL structure(s) (**Table 1**). Aichi virus is predicted to form three SLs within the first 120 nts of its genome, with the first SL (SL-A) being important for virus replication (Sasaki et al., 2001). *Cre* (*cis*-acting element) has been shown to be critical for replication of positive-strand RNA viruses (Pogue and Hall, 1992; Pogue et al., 1994; McKnight and Lemon, 1998; Mason et al., 2002). In *Picornaviridae*, *cre* elements can be found within the protein-coding region of the genome of rhinoviruses (McKnight and Lemon, 1998), enteroviruses (Goodfellow et al., 2003; Paul et al., 2003; Rieder et al., 2003), cardioviruses (Lobert et al., 1999), or within the noncoding regions (Mason et al., 2002). For example, the 5′-UTR of FMDV contains a short (S) hairpin loop *cre* structure upstream of the IRES. The FMDV *cre* was demonstrated to be essential for RNA genome replication (Mason et al., 2002, 2003), and it was also shown to function in *trans* (Tiley et al., 2003). The *cre* hairpin has a conserved AAACA sequence in the apical loop region that is also present in genomes of other picornaviruses (Steil and Barton, 2009).

Picornaviruses recruit various host proteins to mediate viral translation and RNA replication. For example, the PV cloverleaf binds PCBP1 and PCBP2, which facilitates its interaction with the viral 3CD protein (Gamarnik and Andino, 1997), and in the case of PCBP2, is required for both translation and viral RNA synthesis initiation in infected cells (Walter et al., 2002). Moreover, cleavage of PCBP2 by the PV 3CD proteinase contributes to viral translation inhibition. The 5′-UTR of enterovirus 71 (EV71) interacts with hnRNP K protein, which is important for viral replication (Lin et al., 2008). The cloverleaf and the IRES of coxsackievirus B3 (CVB3) are known to interact with PTB-associated splicing factor (PSF) (Dave et al., 2017). FMDV 5′-UTR is known to associate with RNA helicase A (RHA), and that interaction impacts the life cycle of the virus (Lawrence and Rieder, 2009). Interestingly, during the course of FMDV infection, RHA co-precipitated with viral proteins 2C and 3A as well as cellular PABP, shown in close proximity to each other via immunofluorescent microscopy (Lawrence and Rieder, 2009). Also, the IRES of picornaviruses interacts with La, Sam68 (68 kDa Src-associated protein in mitosis), PTB, and Srp20 host proteins (Hunt and Jackson, 1999; Ray and Das, 2002; Bedard et al., 2007; Lawrence et al., 2012; Rai et al., 2015), which improves translation. Upstream of N-ras (Unr) RNA-binding protein is required for initiation of IRES-driven translation in human rhinovirus and PV (Hunt et al., 1999; Boussadia et al., 2003). On the other hand, interactions of AUF-1, Gemin5, and FBP-2 with the 5′-UTR of picornaviruses negatively regulate translation (Lin et al., 2009; Cathcart et al., 2013; Piñeiro et al., 2015; Francisco-Velilla et al., 2016). The functional significance of these interactions for the progression of infection in host cells is still not fully understood.

The 3′-UTR of picornaviruses is much shorter compared to the 5′-UTR and it is most often in the range of 100–300 nts. Some picornaviruses, however, have unusually S or long (L) 3′-UTRs. Kunsagiviruses have a 3′-UTR that consists of around 25 nts (Boros et al., 2013), while the 3′-UTR of a rosavirus is predicted to be 795 nts in length (Phan et al., 2011). Although a SL is a characteristic feature of the *Picornaviridae* 3′-UTRs, the length and the number of these loops vary among species (**Figures 1E–H** and **Table 1**). For example, the feline sakobuvirus has only one predicted SL (Ng et al., 2014), FMDV has two separate SLs (Serrano et al., 2006), and mengovirus is predicted to form three SLs (Duque and Palmenberg, 2001). The SLs of different viruses can form additional arrangements; for example, the two 3′-UTR SLs of Seneca Valley virus form a “kissing loop,” shown to be important for enterovirus replication (Mirmomeni et al., 1997; Hales et al., 2008) (**Figure 1F**). Some *Picornaviridae* genera – such as Kobuvirus, Gallivirus, Sakobuvirus, or Sicinivirus – have a characteristic “barbell” shape of a SL (**Figure 1G**). The precise function of this structure is unknown, although it was suggested to be important for viral replication (Boros et al., 2012). Similar to the 5′-UTR, the secondary structures within the 3′-UTR are important for picornavirus replication, and virus-induced pathogenesis (Merkle et al., 2002). In PV, the circularization of the viral genome and replication depend on binding of host proteins to the 3′-UTR (Herold and Andino, 2001). In FMDV, evidence for distant RNA–RNA interactions between the 3′-UTR and the 5′-UTR has been suggested, as well as for the 5′-terminus S fragment binding to PCBP and p47 cellular proteins (Serrano et al., 2006).

Recent studies suggest that multiple highly ordered secondary structures are present throughout the genome of picornavirus and related RNA viruses including protein-coding region (Mauger et al., 2015; Pirakitikulr et al., 2016; Logan et al., 2017) and UTRs, in addition to those described earlier. Logan et al. (2017; Pirakitikulr et al., 2016) identified putative packaging signals (PPSs) in RNA structural motifs of 5′-UTR and ORF that play a role in packaging of viral RNA genome in a capsid of picornaviruses. Atomic-scale resolution of bimolecular structure in native state due to recent advances in cryo-electron microscopy, and new computational and laboratory tools, could uncover novel physical and functional aspects of RNA structural elements (Hesketh et al., 2015; Pirakitikulr et al., 2016).

## Viral Infection vs. the Innate Immune Response: a Fight for Dominance

Once a picornavirus enters a host cell and starts amplifying its genome, the host defense mechanisms activate the immune pathways to combat the invader. Although both arms of the immune system, innate and acquired, are ultimately needed to fend off a viral infection and prevent future outbreaks, the innate immune system is the first line of defense. Behind this remarkable response lies an orchestrated sequence of events responsible for recognition of the invaders, initiation of intracellular signaling cascades, and activation of the acquired immunity, all of which are crucial for the establishment of an antiviral state in the host. The recognition of a pathogen is of great importance, since a host can only initiate an antiviral immune response once it detects non-self entities. Pathogen-associated molecular patterns, or simply PAMPs, are small pieces of viral genome that can be single-stranded (ssRNA) – representing either a part of a viral genome, or a viral replication product – or double-stranded (dsRNA). These conserved structures are recognized by PRRs: Toll-like receptors (TLRs), NOD-like receptors (NLRs), and RIG-I-like receptors (RLRs) (Takeuchi and Akira, 2009). Following the recognition of viral particles, a cascade of molecular events activates downstream components of the innate immune system. For example, the TLR family members activate IFN-β signaling via TIR-containing adaptors, such as myeloid differentiation primary response 88 (MyD88) (Takeda and Akira, 2005) and they also mediate NF-κB activation (Jiang et al., 2004). TLRs – RIG-I and MDA5 – interact with MAVS to activate IFN type I (Takeuchi and Akira, 2009). NLRs can initiate the response of the innate immune system by interacting with the apoptosis-associated speck-like protein (ASC), which leads to induction of IL-1β and IL-18 (Keller et al., 2008; Lu et al., 2014).

Picornaviruses are thought to trigger the innate immune response primarily via an MDA-5 receptor (Kato et al., 2006). This well-studied MDA-5 function is thought to be accomplished through recognition of L dsRNA fragments, representing either a part of a viral genome or a viral replication product (Kato et al., 2006; Feng et al., 2012). The generation of the minus (-) RNA strand, and the formation of dsRNA, known as the replicative form (RF), triggers a significant IFN α/β response (Feng et al., 2012). Furthermore, purified picornavirus RF is capable of binding to, and activating, the MDA-5 receptor *in vitro* (Feng et al., 2012). Experiments described by Pichlmair et al. (2009) suggest that structural features, such as branched dsRNA, may also contribute to MDA-5 activation. Nonetheless, it has been shown that other PRRs are also affected during a picornavirus infection. For instance, RIG-I is degraded during EMCV infection (Papon et al., 2009) and expression of full-length RIG-I reduces EMCV replication (Yoneyama et al., 2004). Cleavage of RIG-I during infection has been described for other members of the *Picornaviridae* family, such as PV, rhinovirus types 1a and 16, and echovirus type 1 (Barral et al., 2009a). Furthermore, mice lacking TLR-3 have higher mortality rates than wild-type mice after PV infection (Abe et al., 2012). Picornaviruses have also developed a variety of mechanisms to subvert the host adaptive and innate responses. They include: (i) degradation of cytoplasmic sensors that induce IFN expression (Barral et al., 2007; Barral et al., 2009a,b), (ii) inhibition of protein secretion affecting IFN and other cytokines (Doedens et al., 1997; Dodd et al., 2001; Neznanov et al., 2001; Choe et al., 2005), and (iii) inhibition of antigen presentation in the context of major histocompatibility complex (MHC) class I molecules, which impairs the cytotoxic T-cell (CTL) response (Deitz et al., 2000).

## 5′- and 3′-Viral UTR Rnas Influence the Course of Infection

Why would viruses maintain long, noncoding regions that fold into complex, secondary structures? From the standpoint of viral replication, this would seem counterintuitive as it could increase the time needed to synthesize a single viral molecule and potentially delay infection. On the contrary, recent studies strongly suggest that the viral noncoding regions can help evade the host immune system. In particular, specific motifs and/or secondary structures contained in these regions may hold a key to viral pathogenicity.

**FIGURE 2:**
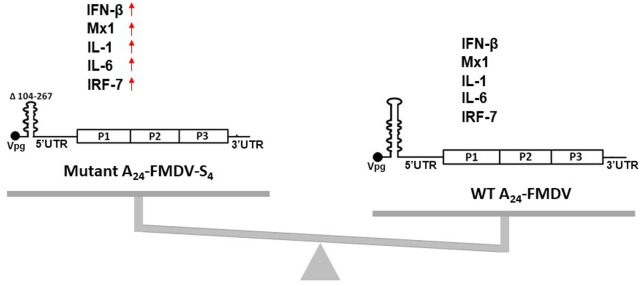
Schematic diagram comparing the activation of the innate immune system in host cells infected with either a WT A_24_-FMDV virus or an A_24_-FMDV-S_4_ mutant virus that contains a 164-nt deletion within the S fragment. The predicted S fragment loops of each virus are depicted as drawings and the upregulated genes of the innate immune system are indicated using red arrows.

One of the most recent pieces of evidence that emphasized the importance of 5′-UTRs in the activation of the host immune system comes from work on FMDV, a member of the Aphthovirus that causes acute disease in cloven-hoofed animals. Its 5′-UTR consists of a S fragment, followed by a poly(C) track, a L fragment made up of PKs, *cre*, and IRES (Mason et al., 2003; Carrillo et al., 2005). Newer findings revealed that the 360 bp hairpin-like loop, called the S fragment, plays a role in innate immunity (Kloc et al., 2017). Kloc et al. (2017) engineered 13 deletions from the upper part of the S fragment hairpin loop and suggested a correlation between the extent of the deletions and the ability to initiate an innate immune system response. Deleting over 164 nts from the upper part of the S fragment loop of the virus enhanced the activation of IFN-β, ISGs, and pro-inflammatory cytokines *in vitro* (**Figure 2**). In the context of an infectious virus, the mutant virus carrying the S fragment 164 nts deletion was attenuated in mice and these immunized animals were fully protected against the challenge with the wild-type FMD virus (Kloc et al., 2017). Importantly, all generated viruses that had 164 nts, or fewer, deleted from the upper part of the S fragment loop were viable, which is in agreement with naturally occurring S fragment FMDV variants of serotypes O, C, and A. In fact, Valdazo-González et al. (2013) described an FMDV isolate with a 70-nt deletion in the S fragment located at positions 148–217 of O/HKN/15/2010. Serotype A isolates found in Argentina in 1959 and 1961 (Carrillo et al., 2005), serotype C isolate from the United Kingdom (Carrillo et al., 2005), and serotype A viruses from India found in 2009 (Subramaniam et al., 2011), all have deletions in the S fragment upper-loop, yet they remain infectious. This evidence strongly suggests that the S fragment of the 5′-UTR can tolerate some deletions without compromising pathogenicity. Furthermore, *in vitro* generated RNA transcripts of the full S fragment induced antiviral state in cell culture and *in vivo* (Rodríguez-Pulido et al., 2011a,b; Kloc et al., 2017). Interestingly, *in vitro* produced S4 RNA transcript, containing the 164 nts deletion, induced higher expression levels of selected innate immune response genes than the full-length S fragment, further reinforcing the importance of the upper-loop of the S fragment in immune system response. Evidence suggests that the virus with the shorter S fragment upper-loop carries a different molecular signature that could make it more susceptible to induction of an innate immune response in the host cell. It is not possible to exclude that viral or host proteins that may bind to the upper-loop of the S fragment are involved in these phenomena. In fact, FMDV, like other members of the *Picornaviridae* family, is known to depend on 5′-UTR–host protein interactions for viral replication, translation, and pathogenesis (Walter et al., 2002; Perera et al., 2007; Lawrence and Rieder, 2009). New studies will be necessary to shed light on the mechanism behind an enhanced innate immune response to the FMDV virus with the shortened S fragment loop.

The 3′-UTR of FMDV also plays a role in the activation of innate immunity. Composed of two SLs and a poly(A) track (Belsham, 2005), the 3′-UTR is important for both viral replication and virulence (García-Nuñez et al., 2014). *In vitro* transcribed 3′-UTR elicits a potent IFN-β response in transfected porcine cells, while deleting 3′-UTR from the FMDV genome diminishes IFN-β induction (Rodríguez-Pulido et al., 2011a). Importantly, disrupting the secondary structure of 3′-UTR negatively affects the immune response (Rodríguez-Pulido et al., 2011a), suggesting that the secondary structures of the noncoding regions could act as PAMP motifs.

Evidence gathered in Enteroviruses suggest that their 5′-UTRs contribute to the establishment and/or maintenance of persistent infection. Enteroviruses constitute one of the most common human pathogens and are responsible for many diseases, including respiratory infections, poliomyelitis, myocarditis, hand-foot-and-mouth disease, aseptic meningitis, or hemorrhagic conjunctivitis. Although still controversial, persistent enterovirus infections have been associated with chronic diseases, such as type I diabetes, chronic myocarditis, and dilated cardiomyopathy (Chapman and Kim, 2008; Bopegamage, 2016). The 5′-UTR of enteroviruses contain secondary structure domains, where cloverleaf constitutes domain I, while depending on enterovirus genome, IRES occupies domains II–VI (Nicholson et al., 1991) or II–VII (Bailey and Tapprich, 2007).

Coxsackievirus B, a member of the Enterovirus genus, is known to cause myocarditis, which, in its acute form, can result in dilated cardiomyopathy and even lead to death (Mason, 2003). It has been shown that CVB2 viral RNA containing 5′-terminal deletions can be detected in mouse and human cardiac tissue in the absence of cytopathic effect (CPE) (Kim et al., 2005; Chapman et al., 2008; Smithee et al., 2015). Specifically, the loss of nts 1–22 and 1–25 of stem within the CVB2 cloverleaf was reported in heart tissues coming from a fatal case of myocarditis patients (Oka et al., 2005). Restoration of the deleted nucleotides in a CVB3/5NTRMito construct reestablished the lytic phenotype *in vitro* and caused myocarditis in animal studies (Chapman et al., 2008), which strongly suggests the importance of the 5′-UTR regions for viral pathogenicity. It is intriguing that persistent populations of CVB3 containing 15–48 deletions in the 5′-UTR were also reported in human endomyocardial tissues (Bouin et al., 2016). Specifically, Bouin et al. (2016) suggested that these low replicative 5′-UTR deletions may cause persistent human cardiac infections and potentially help a wild-type CVB3 virus infect the host by genomic recombination processes (Holmblat et al., 2014; Bouin et al., 2016). A murine model revealed that sequences within the 5′-UTR SL II of CVB3 are determinants of cardiovirulence and contribute to CVB3-induced heart disease (Dunn et al., 2000, 2003). Furthermore, Beaulieux et al. (2005) developed a model system of echovirus 6 chronic infection and revealed two mutations in the 5′-UTR 6 months after the start of the infection: a single mutation at nt 30 in the cloverleaf and a mutation at 108 nts upstream of domain II of IRES. It is thought that these 5′-UTR mutations may help establish a persistent disease state, although the exact mechanism needs to be elucidated.

Specific sequences in the 5′-UTR may be critical in the host recognition of the viral genome, which is crucial for triggering an immune response. Point mutation C97U in the 5′-UTR of CVB2 has been shown to minimize myocarditis in a mouse model system (Massilamany et al., 2015). Previous studies have also shown the importance of nucleotide variations in the 5′-UTR, particularly U-to-C substitution at position 234, in decreasing cardiovirulence in mice (Chapman et al., 1997). Although this hypothesis needs to be investigated, it is also possible that the deletions and/or point mutations described in these studies affect the secondary structures of domain I CVB cloverleaf and/or contribute to the ability of the virus to escape the immune responses of the host. Alternatively, these mutations may disrupt viral/host protein binding, which could be important for establishing infection.

Experimental evidence suggests that the 3′-noncoding region of enteroviruses is important for viral pathogenicity, induction of host regulatory immune response, and translation rate at the IRES (Dobrikova et al., 2003; Lin and Shih, 2014). The enterovirus 3′-UTR typically folds into two SL structures, referred to as domains X and Y (Pilipenko et al., 1992) (**Table 1**). However, CVB3 and other members of the human enterovirus B family contain an additional SL domain Z (SLD Z), which, along with domain Y, can form a second superhelical domain (Merkle et al., 2002) (**Table 1**). Deleting the SLD Z out of CVB3 does not impact viral growth in cell culture, but diminishes the ability of the virus to cause myocarditis and pancreatitis (Merkle et al., 2002). While it is possible that the reduced virulence may be caused by lower tissue-specific replication of the SLD Z deletion virus, the potential induction of the innate immune system in the absence of the SLD Z domain has also been proposed. A similar phenomenon was described in human EV71 (HEV71), known to cause hand–foot-and-mouth disease – a common infection in children under 5 years old – characterized by sores in the mouth and blisters on hands and feet. Viruses that lack 17 nts from the proximal part of the 3′-UTR of HEV71 have normal viral RNA synthesis and translation, but produce small plaques and diminished viral titers during infection at low MOI (Kok et al., 2012). Future tests in an animal model system may reveal if these 3′-UTR deletion viruses cause disease *in vivo*.

Cardioviruses, known to infect many mammalian species, have been linked to myocarditis, type I diabetes, encephalitis, neurological diseases, and multiple sclerosis-like symptoms (Carocci and Bakkali-Kassimi, 2012). Studies in mengovirus, a member of encephalomyocarditis virus (EMCV) species, suggest the involvement of noncoding RNA in virulence. Duque and Palmenberg (2001) engineered a precise deletion of the entire SL I of the 3′-UTR and showed that it is nonessential for viral growth. An infectious virus lacking SL I grows to similar titer levels as the parental mengovirus and it has similar RNA synthesis and translation profiles. Yet, most animals infected with the deletion virus survive, or are only partially paralyzed, which is contrary to wild-type virus infection (Duque and Palmenberg, 2001). This evidence strongly suggests the involvement of the mengovirus 3′-UTR in neurovirulence. Deleting the entire SL I may affect the secondary RNA structure of the 3′-UTR, which could be responsible for the described phenotype. It is also possible that the missing sequences may cause rearrangements, or loss of potential virus or host protein binding within the region.

## Concluding Remarks and Future Directions

The innate immune response is the first line of host defense against infection and it is activated rapidly after exposure to pathogen. Investigating how pathogen-derived molecules activate the innate immune responses has been an active area of research since the concept of PAMPs was first proposed. Work in this field has only begun to explain how viruses have developed methods to evade the innate immune system response. In this review, we have discussed evidence in *Picornaviridae* that suggests the importance of viral noncoding genomic elements, or specific sequences or structures associated with them, in escaping the innate immune system response or contributing to the establishment of persistent infection state. In this respect, it is important to recognize that particular viral 5′- and 3′-UTR motifs may have been selected for during the course of evolution to help ensure infection.

A recent study revealed that a virus can alter its structural RNA elements to avoid recognition by the host (Hyde et al., 2014). IFIT – an IFN-induced gene – is a cytosolic viral sensor that detects viral 5′ppp RNA, which helps decrease viral replication and consequently affects viral pathogenesis (Pichlmair et al., 2011; Fensterl and Sen, 2015). In Alphaviruses, the secondary structural motifs in the 5′-UTR counteract the antiviral activity of IFIT (Hyde et al., 2014), which helps evade the recognition of the immune system and establish infection. Although it remains to be determined how the secondary structure or specific sequence of the Alphavirus 5′-UTR may affect binding to IFIT, it is interesting to speculate that other viruses may also rely on their noncoding RNA elements to escape the activation of the immune system. The S fragment of the 5′-UTR of FMDV, described earlier in the review, confirms this hypothesis. Viruses with an artificially diminished S fragment loop trigger an enhanced innate immune system response and do not develop disease in a mouse model system, suggesting that a portion or a specific sequence of the loop may help the virus evade the host immune system.

Much remains to be learned about the strategies used by viruses to improve their chances of infection and the molecular mechanism behind these phenomena. Research on the role of the noncoding regions in viral pathogenicity may help in the development of novel antiviral drugs and vaccine strategies. In this regard, defined structural domains that belong to 5′- or 3′-FMDV UTRs elicit an upregulated immune response in mice and swine cells, and reduce the probability of subsequent infections. Thus, these viral elements can be used as adjuvants to currently available vaccine strategies, boosting the effectiveness of the vaccine, or enhancing protection. As the result, further investigation of the noncoding RNAs may open up new avenues in antiviral research.

## Author Contributions

All authors listed have made a substantial, direct and intellectual contribution to the work, and approved it for publication.

## Conflict of Interest Statement

The authors declare that the research was conducted in the absence of any commercial or financial relationships that could be construed as a potential conflict of interest.
